# Variation in conserved non-coding sequences on chromosome 5q and susceptibility to asthma and atopy

**DOI:** 10.1186/1465-9921-6-145

**Published:** 2005-12-10

**Authors:** Joseph Donfack, Daniel H Schneider, Zheng Tan, Thorsten Kurz, Inna Dubchak, Kelly A Frazer, Carole Ober

**Affiliations:** 1 Department of Human Genetics, 920 E. 58^th ^Street, The University of Chicago, Chicago, IL 60637, USA; 2 Perlegen Sciences, Mountain View, CA 94043, USA; 3 Lawrence Berkeley National Laboratory, Berkeley, CA 94720, USA

## Abstract

**Background:**

Evolutionarily conserved sequences likely have biological function.

**Methods:**

To determine whether variation in conserved sequences in non-coding DNA contributes to risk for human disease, we studied six conserved non-coding elements in the Th2 cytokine cluster on human chromosome 5q31 in a large Hutterite pedigree and in samples of outbred European American and African American asthma cases and controls.

**Results:**

Among six conserved non-coding elements (>100 bp, >70% identity; human-mouse comparison), we identified one single nucleotide polymorphism (SNP) in each of two conserved elements and six SNPs in the flanking regions of three conserved elements. We genotyped our samples for four of these SNPs and an additional three SNPs each in the *IL13 *and *IL4 *genes. While there was only modest evidence for association with single SNPs in the Hutterite and European American samples (P < 0.05), there were highly significant associations in European Americans between asthma and haplotypes comprised of SNPs in the *IL4 *gene (P < 0.001), including a SNP in a conserved non-coding element. Furthermore, variation in the *IL13 *gene was strongly associated with total IgE (*P *= 0.00022) and allergic sensitization to mold allergens (*P *= 0.00076) in the Hutterites, and more modestly associated with sensitization to molds in the European Americans and African Americans (*P *< 0.01).

**Conclusion:**

These results indicate that there is overall little variation in the conserved non-coding elements on 5q31, but variation in *IL4 *and *IL13*, including possibly one SNP in a conserved element, influence asthma and atopic phenotypes in diverse populations.

## Background

Comparison of human DNA sequences with those of other mammalian species is a powerful method for identifying functionally important sequence elements in the human genome because sequences with function tend to be evolutionarily conserved whereas those without function tend to accumulate variation over time. In fact, ~50% of the DNA sequences that are evolutionarily conserved between humans and mice lie outside of coding sequences of known genes [1]. Some of these conserved non-coding sequences have been shown to be long-range transcriptional regulatory elements participating in the temporal and tissue-specific expression patterns of genes [2,3].

Previous comparison of a 1 Mb region on human chromosome 5q31, which includes the cytokine genes encoding the T-helper 2 (Th2) cytokines, interleukin (IL)-4, IL-5, and IL-13, with the syntenic murine segment identified highly conserved non-coding sequences [4]. Examination of these conserved non-coding sequences in five additional mammalian species demonstrated that these elements are frequently conserved in all mammals. The longest conserved non-coding sequence, called CNS-1, is located between the *IL4 *and *IL13 *genes and showed a high degree of conservation across species [4]. Functional evaluation of CNS-1 in mutant mice revealed its role in the control of the global expression of *IL4*, *IL5 *and *IL13*, suggesting that CNS-1 acts as a coordinate regulator of these three genes [4,5].

This interval on human 5q31 is particularly intriguing because in addition to housing a cluster of genes encoding many Th2 cytokines, linkage to this region has been demonstrated with asthma-related phenotypes in at least six different populations [6-11]. Moreover, variation in the promoter, -589C/T (also referred to as -590C/T) [12], intron 2, +3017G/T [13], and 5'-untranslated region (UTR), +33C/T [14], of the *IL4 *gene and in the promoter, -1112C/T (also referred to as -1055C/T) [15], and coding region, Arg130Gln (also referred to as Arg110Gln) [16,17], of the *IL13 *gene have been associated with asthma and atopic phenotypes in many studies (reviewed in ref[18]. However, the specific variation that underlies the linkages described above has not been identified (reviewed in ref. [19].

It is likely, therefore, that additional variation in this interval contributes to susceptibility to both asthma and atopic phenotypes. In the present study, we screened six non-coding elements on 5q31 that are evolutionarily conserved between the human and murine genomes and are thus possible regulatory elements. We studied 10 polymorphisms across this region, including two within and two flanking conserved non-coding elements, and evaluated their relationship to asthma and atopy in members of a large Hutterite pedigree and in well-defined African American and European American patient populations.

## Methods

### Sample composition

**Figure 1:**
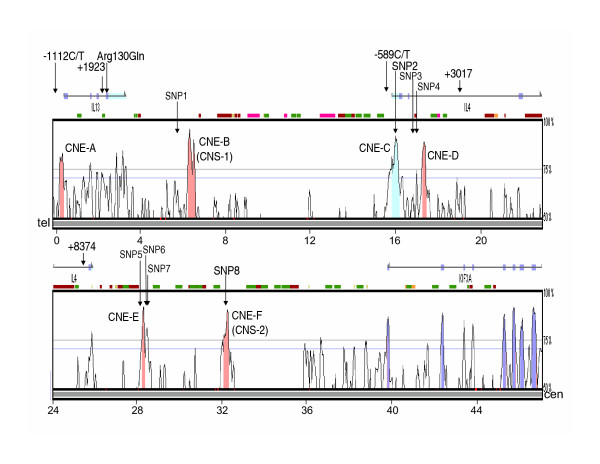
VISTA plot [24] displaying evolutionarily conserved sequences identified by the comparison of ~48 kb of human 5q31 DNA encoding the *IL4, IL13 *and *KIF3A *genes with murine sequences (BAC clone AF276990). On the horizontal axis, conserved sequences are plotted in relation to their position in the human reference sequence; kb distances are shown under the horizontal bar. The height of the peaks on the vertical axis indicates the level of conservation in percent identity between the human reference sequence and the murine sequences. Conserved sequences (>100 bp and >70% identity) defined as coding exons (dark blue), untranslated exons (light blue) and non-coding (red) are shown. The exons in each of three genes are shown as rectangle boxes; only the 3' end (exons 9 through 16) of *KIF3A *is shown. Six conserved non-coding elements were examined in this study (CNE-A - CNE-F). The SNPs identified or genotyped in this study and their approximate locations are shown. CNE-B corresponds to CNS-1 and CNE-F corresponds to CNS-2 described by Loots et al. [4].

Conserved non-coding elements (Figure 1) were screened for SNPs in DNA from 10 African American and 10 European American unrelated controls, and from 10 individuals who are members of a founder population, the Hutterites. The 10 Hutterites were selected to represent distant branches of their pedigree but without regard to disease status.

Associations with asthma and atopy were evaluated in a large Hutterite pedigree [9] and in outbred individuals ascertained in Chicago. Six hundred thirty eight Hutterites were evaluated for asthma and atopy, as previously described [9]; 71 had a diagnosis of asthma, 156 were bronchial hyperresponsive to methacholine, and 311 were atopic. The Chicago samples included 205 African Americans and 126 European Americans with asthma and 388 control subjects with a negative personal and family history of asthma (183 African Americans and 205 European Americans). Subjects included in this study reported having had at least three grandparents who were either of African American or European ancestry. Given the allele frequencies observed in these samples (Table 4), we had 80% power to detect a relative risk of ≥ 1.7 in the African Americans and ≥ 2.2 in the European Americans [20].

### Evaluation of phenotypes

The Hutterites were evaluated for asthma and atopy using previously described protocols [9]. Exposure to cigarette smoke among the Hutterites was rare. The 331 unrelated asthma cases were recruited in Chicago as part of the Collaborative Study on the Genetics of Asthma (CSGA) and met the same diagnostic critieria as that used for the Hutterites [21,22]. Subjects with a history of cigarette smoking (>3 pack-year equivalent) were excluded from these studies. Atopy was defined by skin prick test. No clinical testing was performed on the control subjects. These protocols were approved by The University of Chicago Institutional Review Board; written consent was obtained from all subjects.

### Identification of conserved sequences

An ~40 kb interval on human 5q31 was compared to the syntenic region in the mouse using AVID alignment programs [23] and visualized as a VISTA plot [24]. Conserved non-coding sequences were defined as having every contiguous subsegment of length 100 bp to be ≥ 70% identical to its paired sequence. These regions differ slightly from the earlier study [4] because in that study the CNE calculation was made using PIPMaker and here we used VISTA, which was developed after the Loots study.

### Identification of polymorphisms

Amplified PCR products that included the conserved non-coding elements (Additional File, Table 1) were screened for polymorphisms by denaturing high performance liquid chromatography (DHPLC) [25], which detects nearly 100% of mutations in fragments of 600 bp or less [26-29]. PCR products with variant DHPLC patterns were sequenced; the complement of human BAC clone AC004039.1 was used as the reference sequence for identifying SNPs.

**Table 1:** Distribution of SNPs identified in screening sets by ethnic group. The numbers show how many individuals in each group of 10 with the minor allele. AA = African American, EA = European American, HT = Hutterites.

**CNE**	**Location in Bac clone AC004039.1**	**SNP**	**Location in Bac clone AC004039.1**	**Population**		
				
				AA	EA	HT
CNE-A	48566–48741	-	-	-	-	-
		SNP1-C/T	43038	3	0	0
CNE-B^	42346–42674	-	-	-	-	-
CNE-C	32694–33033	SNP2-C/T	32711	6	3	3
		SNP3-C/T	31971	1	0	0
		SNP4-G/A	31695	1	3	3
CNE-D	31406–31590	-	-	-	-	-
		SNP5-T/C	21794	1	0	0
CNE-E	21595–21737	-	-	-	-	-
		SNP6-C/T	21432	4	6	1
		SNP7-G/A	21425	4	6	1
CNE-F*	17615–17863	SNP8-G/C	17713	2	4	1

*Corresponds to CNS-2 [4]

^Corresponds to CNS-1 [4]

### Genotyping

The genotyping methods used in this study are described in Additional File Table 2. In addition to four SNPs in or flanking conserved sequences, we genotyped six known SNPS in the *IL4 *and *IL13 *genes to evaluate LD patterns between these genes and the CNEs and evaluate the relative magnitude of their effects. These SNPs were *IL13*_-1112C/T [15], *IL13*_+1923 [17], *IL13_*Arg130Gln (A/G) [16,17], *IL4*_-589C/T [12], *IL4*_+3017 [13], and *IL4*_+8374A/G (previously identified in our lab).

**Table 2:** 10-SNP haplotype frequencies in the Hutterites. Haplotypes were constructed manually (see Methods). Only individuals with complete haplotype information for both chromosomes are included (N = 1168 chromosomes). SNPs in the *IL13 *gene that are associated with IgE and +SPT in the Hutterites are in bold font.

		*IL13*			*IL4*					Intergenic Region	
		
Haplotype	Frequency	-1112C/T	+1923C/T	Arg130Gln (G→ A)	-589C/T	SNP2C/T	SNP4G/A	+3017G/T	+8374A/G	SNP7A/G	SNP8C/G
1	0.660	C	C	G	C	C	G	G	A	A	G
2	0.070	**T**	C	G	C	C	G	G	A	A	G
3	0.008	C	**T**	**A**	C	C	G	G	A	A	G
4	0.021	**T**	**T**	**A**	C	C	G	G	A	A	G
5	0.021	C	C	G	C	C	G	T	A	G	C
6	0.034	**T**	**T**	**A**	C	C	G	T	A	G	C
7	0.019	C	**T**	**A**	C	C	G	T	A	G	C
8	0.068	C	C	G	T	T	A	T	G	G	C
9	0.098	**T**	**T**	**A**	T	T	A	T	G	G	C

### Statistical analysis

In the Hutterites, genotyping errors were detected using PEDCHECK [30] and deviations from Hardy-Weinberg equilibrium (HWE) were determined using an application modified to allow for related individuals [31]. To test for associations with SNPs and haplotypes, we used a case-control test developed for large pedigrees, as previously described [32]. Haplotypes comprised of 10 SNPs across the interval were constructed manually by the direct observation of alleles segregating in families. During haplotype construction, missing genotypes were filled in if they could be directly inferred from family data but no inferences were made regarding the haplotype composition when there was more than one possible haplotype. Two locus (pairwise) haplotypes were then generated from the larger 10 SNP haplotypes. We corrected for multiple comparisons using a Bonferonni correction for 4 SNPs and 6 pairwise haplotypes (see Results), and we considered significant *P*-values to be <0.0125 (0.05/4) and <0.00833 (0.05/6), respectively.

Deviations from HWE and differences in allele and genotype frequencies between outbred cases and controls were examined using the program FINETTI [33]. Estimation of haplotype frequencies and testing for associations between cases and controls were conducted using the program FAMHAP [34]; 1,000 permutations were used to assess significance. If empiric *P*-values were <0.001, 10,000 permutations were performed. We used the Bonferonni correction for multiple comparisons (10 SNPs, *P *< 0.005; 45 pairwise comparisons, *P *< 0.0011). This is a conservative correction because these SNPs are not truly independent; some occur in the same gene and some are in LD. On the other hand, we did not correct for the number of phenotypes examined because these are also highly correlated. Within each ethnic group we compared the asthmatic and atopic cases to the non-asthmatic controls.

### Linkage disequilibrium

LD plots were generated in the Chicago samples using publicly available software [35].

## Results

### SNP discovery

Six conserved non-coding sequences were identified in the interval between *KIF3A *and *IL13 *on human chromosome 5q31 (Figure 1). Of note is that none of the exons in either *IL4 *or *IL13 *are conserved between human and mouse, or between human and dog [36]. This is quite unusual (see for comparison, the pattern in *KIF3A*) and suggests possible divergence of function or accelerated rates of evolution of the human IL-4 and IL-13 proteins between humans and mice/dogs.

Eight SNPs, referred to as SNP1-SNP8, were identified within or flanking the six conserved elements (Table 1). One SNP (SNP2) in CNE-C was identical to a previously reported SNP, +33C/T [14], and one SNP (SNP8) was in CNE-F. Six additional SNPs were identified in the sequences flanking CNE-B (SNP1), CNE-D (SNP3, SNP4), and CNE-E (SNP5, SNP6, SNP7). No variation was detected in CNE-B, which corresponds to CNS-1 in the Loots study and was previously shown to coordinately regulate *IL4, IL5 *and *IL13 *[4,5,37]. CNS-F, which corresponds to CNS-2 in the Loots study, harbored one variant (SNP8). We note that SNP4 resides within a conserved element in the *IL4 *gene that was identified by Dubchak and colleagues using human-dog sequence comparisons [36]. Furthermore, other than one rare SNP in Chinese (rs17772853; minor allele frequency 0.01), there is no additional variation in these regions reported in dbSNP [38] or in two previous studies of this region [39,40], suggesting that we identified all common variation in these conserved elements.

A description of the eight SNPs and their distribution among the 30 individuals in the screening sample is shown in Table 1. Three SNPs (SNP1, SNP3 and SNP5) were present only in the African American sample. The remaining five SNPs (SNP2, SNP4, SNP6, SNP7 and SNP8) were present in all three groups. SNP6 and SNP7 were the only variants that appeared to be in perfect LD in all three screening samples. Because so few SNPs were discovered in the conserved non-coding elements and because one of the SNPs (SNP7) fell within a conserved element defined using different criteria in another study [40], we genotyped SNP2, SNP4, SNP7 and SNP8, in addition to three known variants each in *IL4 *and *IL13*.

### Patterns of linkage disequilibrium

Nine haplotypes, comprised of 10 SNPs, were present in the Hutterites (Table 2). Three groups of SNPs were in perfect LD in this founder population: +1923C/T and Arg130Gln in the *IL13 *gene; -589C/T, SNP2C/T, SNP4G/A, and +8374A/G in the *IL4 *gene; and +3017G/T in the *IL4 *gene with intergenic SNP7A/G flanking CNE-E and SNP8G/C in CNE-F. For the remaining analyses in the Hutterites, therefore, we used only one SNP from each of these three LD groups, selecting the one with the most complete genotype information (+1923C/T, SNP2C/T, and +3017G/T), and one SNP that was not in perfect LD with any other SNP (-1112C/T).

**Figure 2:**
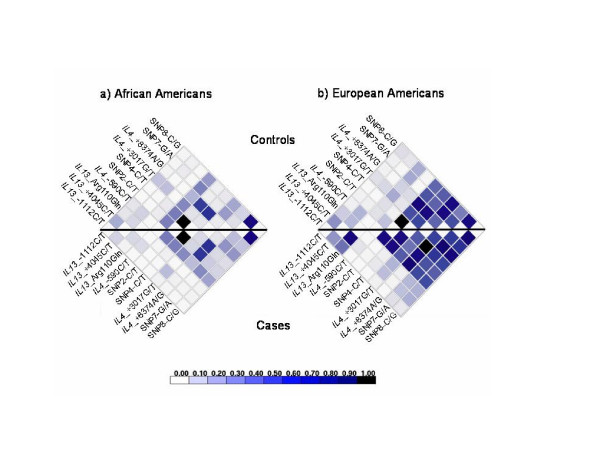
Pairwise LD plots (r^2^) for cases (lower half) and controls (upper half) in a) African Americans and b) European Americans.

The patterns of pairwise LD between the 10 SNPs in the outbred samples are shown in Figures 2a (African Americans) and 2b (European Americans). In both control samples there was little long range LD (r^2 ^≤ 0.30) between SNPs in the *IL4 *and *IL13 *genes and relatively strong LD among SNPs within and in the 3' flanking region of the *IL4 *gene, similar to the pattern in the Hutterites (Table 2). The LD pattern among the African American cases and controls looked similar. However, among the European Americans, there was more LD between the *IL4*_-589 promoter SNP and other variants in the *IL4 *gene in the cases compared with controls. In the controls there was surprisingly little LD between *IL4*_-589 and other *IL4 *SNPs. Because there were few pairs of SNPs that showed perfect LD in the outbred samples and they differed between cases and controls, we analyzed all SNPs in the outbred samples.

### SNP studies in the hutterites

The minor allele frequencies of SNPs in the Hutterites were: *IL13_*-1112T, 0.208; *IL13_*+1923T, 0.173; SNP2-T, 0.156; and *IL4*_+3017T, 0.226. Genotype proportions were in HWE at all loci (*P *> 0.01). In the single SNP analyses, there were modest associations between *IL13_*-1112T and asthma (*P *= 0.025), BHR (*P *= 0.028), and allergic sensitization to CR allergens (*P *= 0.032); and between SNP2-T with allergic sensitization to molds (*P *= 0.034). None of these were significant after correcting for multiple comparisons. However, highly significant associations were present between variation in the *IL13 *gene and sensitization to mold allergens (*lL13_*-1112T, *P *= 0.00067; *IL13_*+1923T, *P *= 0.0074), which remained significant after correcting for multiple comparisons. Moreover, only SNPs in *IL13 *were associated with total serum IgE, with a highly significant association between high IgE and the *IL13_*+1923T allele (*P *= 0.00022) and a more modest association with the *lL13_*-1112T allele (*P *= 0.014). Adjusting for allergic sensitization to molds in the analysis reduced the significance of the *IL13_*+1923T allele, but did not eliminate the association (*P *= 0.0085).

To determine if susceptibility to asthma or atopic phenotypes is determined by combinations of SNPs across this interval or by specific haplotypes, we examined pairwise combinations of the four SNPs (Table 3). Highly significant associations (*P *< 0.001) with +SPT to mold allergens were observed with haplotypes comprised of either SNP in the *IL13 *gene (-1112C/T or +1923C/T) and SNP2 in the *IL4 *gene. Less significant associations were observed with these same pairwise combinations and allergic sensitization to cockroach allergen. However, in all of these analyses, the haplotypes carrying the common alleles at the *IL13 *SNPs (-1112C and +1923C) were underrepresented in the cases compared with controls, and the two haplotypes carrying the minor alleles at the *IL13 *SNPs (-1112T and +1923T) were overrepresented in the cases compared with controls, regardless of the allele at SNP2 (i.e., C or T). Therefore, the results of the haplotype analyses suggest that the *IL13 *SNPs are primarily associated with allergic sensitization to mold and cockroach allergens, and that variation in the *IL4 *gene is not contributing to this association in the Hutterites. None of the haplotypes were associated with asthma, BHR, or the other atopic phenotypes. Thus, in the Hutterites, variation in the *IL13 *gene is strongly associated with total serum IgE and allergic sensitization to mold allergens, and to a lesser extent to cockroach allergens, but not to any of the other phenotypes. None of the SNPs in or near conserved non-coding sequences contributed to susceptibility in the Hutterites.

**Table 3:** Results of 2-SNP haplotype analyses in the Hutterites. In this sample, *IL13*_+1923C/T is in perfect LD (r^2 ^= 1) with *IL13*_Arg130Gln (G→ A); SNP2 C/T is in perfect LD with *IL4*_-589C/T, SNP4G/A, and *IL4*_+8374A/G; IL4_+3017G/T is in perfect LD with SNP7A/G and SNP8C/G (Table 2). Only haplotypes and phenotypes with at least one *P*-value < 0.05 are shown. The number of cases in each analysis is shown in parentheses. *P*-values that were significant after adjusting for multiple comparisons are in bold font.

		Specific IgE Response (+SPT) to	
		
Locus 1	Locus 2	Mold (N = 75)	Cockroach (N = 148)
IL13_--1112	IL13_+1923	**0.0063**	0.042
	SNP2	**0.00020**	**0.0022**
	IL4_+3017	0.033	**0.0074**
IL13_+1923	SNP2	**0.00033**	**0.0017**
	IL4_+3017	0.017	0.042

**Table 4:** Allele and genotype frequencies in case and control samples. The number of individuals in each sample is shown in parentheses. Not all individuals in the samples were genotyped for all SNPs. Only SNP2-T showed a modest association with allergic sensitization to mold allergens in European Americans (*P*_uncorrected _= 0.04).

	*European Americans*				*African Americans*			
SNP	All Asthma	+SPT Any Allergen	+SPT Molds	Controls	All Asthma	+SPT Any Allergen	+SPT Molds	Controls

	(N = 126)	(N = 82)	(N = 46)	(N = 205)	(N = 205)	(N = 160)	(N = 68)	(N = 183)
*IL13_*-1112T	0.259	0.264	0.275	0.213	0.418	0.409	0.375	0.407
CC	0.576	0.586	0.550	0.613	0.337	0.336	0.391	0.360
CT	0.330	0.300	0.350	0.348	0.489	0.510	0.469	0.467
TT	0.094	0.114	0.100	0.039	0.174	0.154	0.140	0.173
*IL13_*+1923T	0.245	0.282	0.238	0.227	0.666	0.664	0.672	0.660
CC	0.575	0.535	0.575	0.584	0.087	0.094	0.094	0.133
CT	0.359	0.366	0.375	0.376	0.495	0.483	0.469	0.413
TT	0.066	0.099	0.050	0.040	0.418	0.423	0.437	0.454
*IL13_*Gln (T)	0.221	0.254	0.272	0.202	0.192	0.191	0.161	0.169
Arg/Arg (C/C)	0.618	0.577	0.625	0.620	0.643	0.639	0.695	0.686
Arg/Gln (C/T)	0.324	0.338	0.325	0.355	0.330	0.340	0.288	0.290
Gln/Gln (T/T)	0.058	0.085	0.05	0.025	0.027	0.021	0.077	0.024
*IL4_*-589T	0.193	0.197	0.163	0.163	0.677	0.678	0.687	0.643
CC	0.670	0.662	0.700	0.702	0.115	0.128	0.125	0.133
CT	0.274	0.282	0.275	0.270	0.397	0.389	0.375	0.447
TT	0.056	0.056	0.025	0.028	0.478	0.483	0.5	0.420
CNE-C_SNP2-T	0.196	0.223	0.257	0.146	0.405	0.419	0.404	0.382
CC	0.667	0.600	0.541	0.730	0.333	0.316	0.333	0.382
CT	0.294	0.354	0.405	0.247	0.524	0.530	0.526	0.471
TT	0.049	0.046	0.054	0.023	0.143	0.154	0.141	0.147
SNP4-A	0.176	0.186	0.187	0.175	0.399	0.412	0.415	0.359
GG	0.692	0.661	0.656	0.675	0.340	0.323	0.321	0.415
GA	0.264	0.305	0.313	0.300	0.525	0.531	0.528	0.452
AA	0.044	0.034	0.031	0.025	0.135	0.146	0.151	0.132
*IL4_*+3017T	0.327	0.339	0.387	0.308	0.850	0.867	0.825	0.805
GG	0.490	0.452	0.382	0.478	0.045	0.061	0.067	0.051
TG	0.367	0.419	0.471	0.429	0.209	0.217	0.217	0.288
TT	0.143	0. 129	0.147	0.093	0.746	0. 727	0.716	0.661
*IL4_*+8374G	0.161	0.164	0.135	0.159	0.275	0.274	0.259	0.257
AA	0.708	0.687	0.730	0.703	0.526	0.533	0.554	0.541
AG	0.261	0.299	0.270	0.275	0.398	0.387	0.375	0.404
GG	0.031	0.014	0	0.022	0.076	0.080	0.071	0.055
SNP7-G	0.279	0.318	0.319	0.317	0.636	0.638	0.614	0.568
AA	0.548	0.485	0.472	0.458	0.156	0.152	0.123	0.213
AG	0.347	0.394	0.417	0.450	0.416	0.421	0.526	0.460
GG	0.105	0.121	0.111	0.092	0.428	0.427	0.351	0.327
CNE-F_SNP8-C	0.323	0.339	0.386	0.293	0.618	0.623	0.598	0.577
CC	0.131	0.113	0.143	0.081	0.393	0.401	0.328	0.335
CG	0.384	0.452	0.486	0.422	0.450	0.444	0.541	0.484
GG	0.485	0.435	0.371	0.497	0.157	0.156	0.131	0.181

### Studies in outbred case-control samples

Allele and genotype frequencies of the 10 SNPs in 337 subjects with asthma and 388 non-asthmatic controls are shown in Table 4 by ethnicity and phenotype. Genotypes were in HWE in the African American and European American control samples (*P *> 0.01). In the single SNP analyses, there was only a modest association between SNP2-T and allergic sensitization to mold allergens in European Americans (*P *= 0.04), which was not significant after adjusting for multiple comparisons.

However, pairwise combinations of SNPs in the *IL4 *gene were significantly associated with asthma and allergic sensitization, primarily in the European American sample (Table 5). In that sample, nearly all of the haplotypes that were associated with asthma and the one most strongly associated with atopy included the *IL4*_-589T allele and other SNPs in the *IL4 *gene (SNP2-T, SNP4-A, *IL4*_+3017T, *IL4*_+8374G, SNP8-C). A haplotype comprised of the *IL13*_+1923T and *IL13*_130Gln alleles was also strongly associated with asthma in this sample. All but one of the seven associations remained significant after adjusting for multiple comparisons. In the African Americans, the frequencies of the *IL13*_-1112T/*IL13*_+1923T and *IL13*_-1112T/*IL4*_+3017T haplotypes were increased in cases with allergic sensitization to mold (*P *= 0.009 and 0.005, respectively), although this was not significant after correcting for multiple comparisons. However, because some of the controls may have been SPT+ to mold allergens, this is a conservative test. Similar to the Hutterites, there were no associations with asthma or SPT to any allergen or with combinations of SNPs in the *IL4 *gene in the African Americans.

**Table 5:** Results of 2-SNP haplotype analysis in a) European Americans cases and controls, and b) African American cases and controls. Empiric *P*-values are based on 1,000 or 10,000 permutations; only haplotypes with at least one *P*-value < 0.05 are shown. *P*-values that were significant after adjusting for multiple (45) comparisons are in bold font; ns, not significant (*P *≥ 0.05).

Locus 1	Locus 2	Asthma vs. Controls	+SPT to Any Allergen vs. Controls	+SPT Molds vs. Controls
**a) European Americans**				
IL13_-1112	IL4_+3017	ns	ns	0.0128
IL13_+1923	IL13_130	**<10**^-4^	0.0011	0.0121
IL13_130	SNP2	ns	0.0295	0.0101
IL4_-589	SNP2	**<10**^-4^	**0.0004**	0.0026
	SNP4	**<10**^-4^	**<10**^-4^	**0.0005**
	IL4_+3017	**0.0003**	0.0030	0.0269
	IL4_+8374	**<10**^-4^	**<10**^-4^	**0.0002**
	SNP7	0.0021	0.0279	0.0335
	SNP8	**0.0002**	0.0072	0.0155
SNP2	SNP4	ns	0.0240	0.0042
	IL4_+3017	ns	0.0131	**<10**^-4^
	IL4_+8374	ns	0.0252	0.0054
	SNP7	ns	ns	0.0019
	SNP8	ns	ns	0.0128
IL4_+3017	SNP7	ns	ns	0.014
SNP7	SNP8	ns	ns	0.014
**b) African Americans**				
IL13_-1112	IL13_+1923	ns	ns	0.009
	IL4_+3017	0.034	ns	0.005

## Discussion and conclusions

Cross-species comparisons are powerful tools for identifying potential functional elements in non-coding DNA [3,4,36,41-44]. However, it is unknown whether conserved non-coding elements in the human genome harbor variation that contributes to inter-individual differences in susceptibility to common diseases. To address this question, we surveyed variation in six conserved non-coding elements in the Th2 cytokine gene cluster on chromosome 5q31 to determine whether such variation, if it exists, is associated with susceptibility to asthma-related phenotypes.

Only one of these conserved non-coding elements, CNS-1 (CNE-B in our study), has been shown to have regulatory properties: the deletion of CNS-1 in transgenic mice results in the reduction of human IL-4, IL-5 and IL-13 producing cells [5,37]. Similar to our results, neither Noguchi et al. [39] nor Banerjee et al. [40] found sequence variation in CNS-1 in 48 individuals of Japanese origin [39] or in 17 individuals of African origin and 23 individuals of European origin [40]. These results combined with ours indicate that CNS-1 is highly conserved among humans and is under strong selective constraints, consistent with its role as a *cis*-acting regulatory element.

CNS-2 (CNE-F in our study) was also among the most conserved non-coding elements identified in a comparison of human 5q31 DNA with conserved syntenic mouse sequences, second only to CNS-1 [4]. We found one SNP in this element (SNP8), similar to the study of Banerjee [40]. However, this variant was not associated with asthma or atopy in the Hutterites or outbred case-control samples. However, we note that SNP8 in CNE-F (CNS-2) is in very strong LD with *IL4*_+3017, which was associated with IgE levels in a previous study in Caucasian subjects [13]. We did not find any variation in CNE-E, although one rare and two common SNPs (SNP5 and SNP6, SNP7, respectively) were identified just outside the boundaries of this element. SNP7 was in a conserved element defined by Banerjee, but this SNP was also not associated with asthma or atopy in our study.

Only SNP2 (+33C/T) in CNE-C was associated with asthma and atopy, and only when considered in combination with other SNPs in the *IL4 *gene. This SNP was previously associated with IgE levels in Japanese (*P *< 0.05) [14]. However, our results indicate that either combinations of SNPs in and near the *IL4 *gene act synergistically to influence susceptibility, or other variation on a haplotype that includes the -589-T, SNP2-T, SNP4-G, +3017-T, +8374-G, and SNP8-C alleles influences susceptibility. In either case, the variation in *IL4 *that influences asthma and atopy resides in non-coding regions. Similarly, the -589-T and +3017-T alleles, which have been associated with asthma and/or atopy in other studies [12,13,45-50], do not by themselves or in combination with each other account for the associations observed in this study.

Lastly, we identified an association between variation in the *IL13 *gene and allergic sensitization to mold allergens in the Hutterites, which was also present, albeit to a lesser degree, in two outbred populations. Associations of other atopic phenotypes with two functional polymorphisms [15,51] in *IL13 *have been reported previously [15-17,52-55], but this is the first report of a specific association with +SPT to molds. Haplotypes comprised of SNPs in the *IL13 *gene were also associated with +SPT to mold allergens in the African American and European American samples, suggesting that either these SNPs interact to confer risk or additional variation in this gene also contributes. In addition, the +1923T and/or 130Gln alleles were also very strongly associated with total serum IgE (as a quantitative trait) in the Hutterites. The association with IgE was only partially accounted for by mold sensitization, indicating a role for this gene in IgE mediated immune responses, consistent with studies in other populations [16,17,54,55].

The fact that we identified associations between variation in the *IL13 *gene and atopy in all three populations (and with asthma in the European Americans), but between variation in the *IL4 *gene and asthma only in the European Americans, reflects the complexity of genetic susceptibility to asthma and atopy. It is notable that allele frequencies at SNPs across this interval differed considerably between the African American and European American samples (Table 4). For example, the minor allele in the European American sample was the more common (major) allele in the African American sample at five loci (*IL13*_+1923, *IL4*_-589, *IL4*_+3017, SNP7, CNE-F_SNP8). At nearly all other loci, the allele frequencies were more even (i.e., closer to 50%) in the African American than in the European American sample. Furthermore, although the overall pattern of LD was similar in the African American and European American control subjects (Figure 2), there was more LD between the -589C/T alleles with alleles at other *IL4 *SNPs in the European American cases compared with controls. The latter is the expected pattern at a disease locus [56], and is consistent with the highly significant associations that we observed between *IL4 *haplotypes and asthma in the European Americans. These differences in allele frequencies and LD patterns may have reduced our power detect associations in the African American sample, particularly with respect to untyped SNPs that might be in LD with *IL4*_-589. Alternatively, the observation that no one SNP or combination of SNPs is penetrant in all populations may reflect the modifying effects of background genes or environmental exposures on risk [57, 58]. This possibility is supported by a genome-wide linkage study of asthma in which different linkage signals were detected in Caucasian and African American families, despite the fact that both groups were evaluated using identical protocols and ascertained at the same centers [10,21]. These results highlight the challenges in elucidating the genetic architecture of complex diseases, which is likely to differ among individuals with different environmental exposures and different genetic backgrounds, some of which is captured by racial/ethnic ancestry.

In summary, these data indicate that the conserved non-coding elements on human chromosome 5q31 in the interval including the *IL13 *and *IL4 *genes do not contain variation that influences disease risk among individuals. SNP2 (+33C/T), in a conserved element (CNE-C) in the *IL4 *gene, may influence susceptibility in combination with other variation in *IL4*, or may merely be in LD with other variation in the gene that influences susceptibility to asthma and atopic phenotypes. Additional studies are required to differentiate between these alternatives, to fully characterize the functional variants in this region that influence disease risk, and to provide a model for understanding the role of non-coding variation on gene function and disease susceptibility.

## List of abbreviations

AA African Americans

CNE conserved non-coding element

CSGA Collaborative Study on the Genetics of Asthma

DHPLC denaturing high performance liquid chromatography

EA European Americans

HWE Hardy-Weinberg equilibrium

IgE immunoglobulin E

LD linkage disequilibrium

*IL4 *interleukin 4

*IL13 *interleukin 13

PCR polymerase chain reaction

SNP single nucleotide polymorphism

SPT skin prick test

Th2 T-helper 2

## Competing interests

The author(s) declare that they have no competing interests.

## Authors' contributions

Dr. Donfack and Mr. Schneider designed primers and performed all of the DHPLC, sequencing and genotyping analyses. Dr. Tan and Dr. Kurz performed the LD studies and all data analyses. Dr. Dubchak and Dr. Frazer performed the VISTA analyses, defined the conserved region, and provided unpublished sequence data. Dr. Ober conceived and designed the study and, with Dr. Donfack, wrote the manuscript. All authors contributed comments to various drafts of the manuscript.

## Supplementary Material

Additional File 1 Table 1. Primer sequences and PCR conditions. Table 2. Genotyping methods. Click here for file

## References

[B1] Frazer KA, Sheehan JB, Stokowski RP, Chen X, Hosseini R, Cheng JF, Fodor SP, Cox DR, Patil N (2001). Evolutionarily conserved sequences on human chromosome 21. Genome Res.

[B2] Nobrega MA, Ovcharenko I, Afzal V, Rubin EM (2003). Scanning human gene deserts for long-range enhancers. Science.

[B3] Boffelli D, Nobrega MA, Rubin EM (2004). Comparative genomics at the vertebrate extremes. Nat Rev Genet.

[B4] Loots GG, Locksley RM, Blankespoor CM, Wang ZE, Miller W, Rubin EM, Frazer KA (2000). Identification of a coordinate regulator of interleukins 4, 13, and 5 by cross-species sequence comparisons.. Science.

[B5] Mohrs M, Blankespoor CM, Wang ZE, Loots GG, Afzal V, Hadeiba H, Shinkai K, Rubin EM, Locksley RM (2001). Deletion of a coordinate regulator of type 2 cytokine expression in mice. Nat Immunol.

[B6] Meyers DA, Postma DS, Panhuysen CIM (1994). Evidence for a locus regulating total serum IgE levels mapping to chromosome 5. Genomics.

[B7] Marsh DG, Neely JD, Breazeale DR, Ghosh B, Freidhoff LR, Schou C, Beaty TH (1995). Genetic basis of IgE responsiveness: relevance to the atopic diseases. Int Arch Allergy Immunol.

[B8] Martinez FD, Solomon S, Holberg CJ, Graves PA, Baldini M, Erickson RP (1998). Linkage of circulating eosinophils to markers on chromosome 5q. Am J Resp Care Med.

[B9] Ober C, Tsalenko A, Parry R, Cox NJ (2000). A second-generation genomewide screen for asthma-susceptibility alleles in a founder population. Am J Hum Genet.

[B10] Xu J, Meyers DA, Ober C, Blumenthal MN, Mellen B, Barnes KC, King RA, Lester LA, Howard TD, Solway J, Langefeld CD, Beaty TH, Rich SS, Bleecker ER, Cox NJ (2001). Genomewide screen and identification of gene-gene interactions for asthma-susceptibility loci in three U.S. populations: Collaborative Study on the Genetics of Asthma. Am J Hum Genet.

[B11] Yokouchi Y, Nukaga Y, Shibasaki M, Noguchi E, Kimura K, Ito S, Nishihara M, Yamakawa-Kobayashi K, Takeda K, Imoto N, Ichikawa K, Matsui A, Hamaguchi H, Arinami T (2000). Significant evidence for linkage of mite-sensitive childhood asthma to chromosome 5q31-q33 near the interleukin 12 B locus by a genome-wide search in Japanese families. Genomics.

[B12] Rosenwasser LJ, Klemm DJ, Dresback JK, Inamura H, Mascali JJ, Klinnert M, Borish L (1995). Promoter polymorphisms in the chromosome 5 gene cluster in asthma and atopy. Clin Exp Allergy.

[B13] Basehore MJ, Howard TD, Lange LA, Moore WC, Hawkins GA, Marshik PL, Harkins MS, Meyers DA, Bleecker ER (2004). A comprehensive evaluation of IL4 variants in ethnically diverse populations: association of total serum IgE levels and asthma in white subjects. J Allergy Clin Immunol.

[B14] Suzuki I, Hizawa N, Yamaguchi E, Kawakami Y (2000). Association between a C+33T polymorphism in the IL-4 promoter region and total serum IgE levels. Clin Exp Allergy.

[B15] van der Pouw Kraan TCTM, van Veen A, Boeije LCM, van Yurl SAP, de Groot ER, Stapel SO, Bakker A, Verweij CL, Aarden LA, van der Zee JS (1999). An IL-13 promoter polymorphism associated with increased risk of allergic asthma. Genes and Immunity.

[B16] Heinzmann A, Mao XQ, Akaiwa M, Kreomer RT, Gao PS, Ohshima K, Umeshita R, Abe Y, Braun S, Yamashita T, Roberts MH, Sugimoto R, Arima K, Arinobu Y, Yu B, Kruse S, Enomoto T, Dake Y, Kawai M, Shimazu S, Sasaki S, Adra CN, Kitaichi M, Inoue H, Yamauchi K, Tomichi N, Kurimoto F, Hamasaki N, Hopkin JM, Izuhara K, Shirakawa T, Deichmann KA (2000). Genetic variants of IL-13 signalling and human asthma and atopy. Hum Mol Genet.

[B17] Graves PE, Kabesch M, Halonen M, Holberg CJ, Baldini M, Fritzsch C, Weiland SK, Erickson RP, von Mutius E, Martinez FD (2000). A cluster of seven tightly linked polymorphisms in the IL-13 gene is associated with total serum IgE levels in three populations of white children. J Allergy Clin Immunol.

[B18] Hoffjan S, Nicolae D, Ober C (2003). Association studies for asthma and atopic diseases: a comprehensive review of the literature. Respir Res.

[B19] Hoffjan S, Ober C (2002). Present status on the genetics of asthma. Curr Opin Immunol.

[B20] Schlesselman JJ (1982). Case-Control Studies.

[B21] CSGA (1997). A genome-wide search for asthma susceptibility loci in ethnically diverse populations. Nature Genetics.

[B22] Lester LA, Rich SS, Blumenthal MN, Togias A, Murphy S, Malveaux F, Miller ME, Dunston GM, Solway J, Wolf R, Samet JM, Marsh DG, Meyers DA, Ober C, Bleecker ER (2001). Ethnic differences in asthma and associated phenotypes: Collaborative Study on the Genetics of Asthma. J Allergy Clin Immunol.

[B23] Bray N, Dubchak I, Pachter L (2003). AVID: A global alignment program. Genome Res.

[B24] Frazer KA, Pachter L, Poliakov A, Rubin EM, Dubchak I (2004). VISTA: computational tools for comparative genomics. Nucleic Acids Res.

[B25] Oefner PJ, Underhill PA (1995). Comparative DNA sequencing by denaturing high-performance liquid chromatography (DHPLC). Am J Hum Genet Suppl.

[B26] Han SS, Cooper DN, Upadhyaya MN (2001). Evaluation of denaturing high performance liquid chromatography (DHPLC) for the mutational analysis of the neurofibromatosis type 1 ( NF1) gene. Hum Genet.

[B27] Lin D, Goldstein JA, Mhatre AN, Lustig LR, Pfister M, Lalwani AK (2001). Assessment of denaturing high-performance liquid chromatography (DHPLC) in screening for mutations in connexin 26 (GJB2). Hum Mutat.

[B28] Roberts PS, Jozwiak S, Kwiatkowski DJ, Dabora SL (2001). Denaturing high-performance liquid chromatography (DHPLC) is a highly sensitive, semi-automated method for identifying mutations in the TSC1 gene. J Biochem Biophys Methods.

[B29] Ravnik-Glavac M, Atkinson A, Glavac D, Dean M (2002). DHPLC screening of cystic fibrosis gene mutations. Hum Mutat.

[B30] O'Connell JR, Weeks DE (1998). PedCheck: A program for identification of genotype incompatibilities in linkage analysis. Am J Hum Genet.

[B31] Bourgain C, Abney M, Schneider D, Ober C, McPeek MS (2004). Testing for hardy-weinberg equilibrium in samples with related individuals. Genetics.

[B32] Bourgain C, Hoffjan S, Nicolae R, Newman D, Steiner L, Walker K, Reynolds R, Ober C, McPeek MS (2003). Novel case-control test in a founder population identifies p-selectin as an atopy-susceptibility locus. Am J Hum Genet.

[B33] http://ihg.gsf.de/linkage/download/finetti.zip

[B34] Becker T, Knapp M (2004). Maximum-likelihood estimation of haplotype frequencies in nuclear families. Genet Epidemiol.

[B35] Dubchak I, Brudno M, Loots GG, Pachter L, Mayor C, Rubin EM, Frazer KA (2000). Active Conservation of Noncoding Sequences Revealed by Three-Way Species Comparisons. Genome Research.

[B36] Lee GR, Fields PE, Flavell RA (2001). Regulation of IL-4 gene expression by distal regulatory elements and GATA-3 at the chromatin level. Immunity.

[B37] Noguchi E, Nukaga-Nishio Y, Jian Z, Yokouchi Y, Kamioka M, Yamakawa-Kobayashi K, Hamaguchi H, Matsui A, Shibasaki M, Arinami T (2001). Haplotypes of the 5' region of the IL-4 gene and SNPs in the intergene sequence between the IL-4 and IL-13 genes are associated with atopic asthma.. Hum Immunol.

[B38] Banerjee P, Bahlo M, Schwartz JR, Loots GG, Houston KA, Dubchak I, Speed TP, Rubin EM (2002). SNPs in putative regulatory regions identified by human mouse comparative sequencing and transcription factor binding site data. Mamm Genome.

[B39] Shamsher MK, Chuzhanova NA, Friedman B, Scopes DA, Alhaq A, Millar DS, Cooper DN, Berg LP (2000). Identification of an intronic regulatory element in the human protein C (PROC) gene. Hum Genet.

[B40] Pennacchio LA, Olivier M, Hubacek JA, Cohen JC, Cox DR, Fruchart JC, Krauss RM, Rubin EM (2001). An apolipoprotein influencing triglycerides in humans and mice revealed by comparative sequencing. Science.

[B41] Rogers HJ, Bate N, Combe J, Sullivan J, Sweetman J, Swan C, Lonsdale DM, Twell D (2001). Functional analysis of cis-regulatory elements within the promoter of the tobacco late pollen gene g10. Plant Mol Biol.

[B42] Koch MA, Weisshaar B, Kroymann J, Haubold B, Mitchell-Olds T (2001). Comparative genomics and regulatory evolution: conservation and function of the Chs and Apetala3 promoters. Mol Biol Evol.

[B43] Walley AJ, Cookson WO (1996). Investigation of an interleukin-4 promoter polymorphism for associations with asthma and atopy.. J Med Genet.

[B44] Kawashima T, Noguchi E, Arinami T, Yamakawa-Kobayashi K, Nakagawa H, Otsuka F, Hamaguchi H (1998). Linkage and association of an interleukin 4 gene polymorphism with atopic dermatitis in Japanese families. J Med Genet.

[B45] Noguchi E, Shibasaki M, Arinami T, Takeda K, Yokouchi Y, Kawashima T, Yanagi H, Matsui A, Hamaguchi H (1998). Association of asthma and the interleukin-4 promoter gene in Japanese. Clin Exp Allergy.

[B46] Sandford AJ, Chagani T, Zhu S, Weir TD, Bai TR, Spinelli JJ, Fitzgerald JM, Behbehani NA, Tan WC, Pare PD (2000). Polymorphisms in the IL4, IL4RA, and FCERIB genes and asthma severity. J Allergy Clin Immunol.

[B47] Burchard EG, Silverman EK, Rosenwasser LJ, Borish L, Yandava C, Pillari A, Weiss ST, Hasday J, Lilly CM, Ford JG, Drazen JM (1999). Association between a sequence variant in the IL-4 gene promoter and FEV(1) in asthma. Am J Respir Crit Care Med.

[B48] Zhu S, Chan-Yeung M, Becker AB, Dimich-Ward H, Ferguson AC, Manfreda J, Watson WT, Pare PD, Sandford AJ (2000). Polymorphisms of the IL-4, TNF-alpha, and Fcepsilon RIbeta genes and the risk of allergic disorders in at-risk infants. Am J Respir Crit Care Med.

[B49] Vladich FD, Brazille SM, Stern D, Peck ML, Ghittoni R, Vercelli D (2005). IL-13 R130Q, a common variant associated with allergy and asthma, enhances effector mechanisms essential for human allergic inflammation. J Clin Invest.

[B50] Liu X, Nickel R, Beyer K, Wahn U, Ehrlich E, Freidhoff LR, Bjorksten B, Beaty TH, Huang SK (2000). An IL13 coding region variant is associated with a high total serum IgE level and atopic dermatitis in the German multicenter atopy study (MAS- 90). J Allergy Clin Immunol.

[B51] Howard TD, Whittaker PA, Zaiman AL, Koppelman GH, Xu J, Hanley MT, Meyers DA, Postma DS, Bleecker ER (2001). Identification and association of polymorphisms in the interleukin-13 gene with asthma and atopy in a Dutch population.. Am J Respir Cell Mol Biol.

[B52] Leung TF, Tang NL, Chan IH, Li AM, Ha G, Lam CW (2001). A polymorphism in the coding region of interleukin-13 gene is associated with atopy but not asthma in Chinese children. Clin Exp Allergy.

[B53] DeMeo DL, Lange C, Silverman EK, Senter JM, Drazen JM, Barth MJ, Laird N, Weiss ST (2002). Univariate and multivariate family-based association analysis of the IL-13 ARG130GLN polymorphism in the Childhood Asthma Management Program. Genet Epidemiol.

[B54] Hayes MG, Tsuchiya T, del Bosque-Plata L, Cox NJ (2004). Patterns of linkage disequilibrium in calpain-10. Am J Hum Genet.

[B55] Marchini J, Donnelly P, Cardon LR (2005). Genome-wide strategies for detecting multiple loci that influence complex diseases. Nat Genet.

[B56] Ober C (2005). Perspectives on the past decade of asthma genetics. J Allergy Clin Immunol.

