# Supplementary material for: Variation in conserved non-coding sequences on chromosome 5q and susceptibility to asthma and atopy
Source: Respir Res. 2005 Dec 10;6(1):145. doi: 10.1186/1465-9921-6-145 (PMC1325232; doi:10.1186/1465-9921-6-145)
Supplement: Additional File 1 — Table 1. Primer sequences and PCR conditions. Table 2. Genotyping methods. [file 1465-9921-6-145-S1.doc]

**Additional Files**

**Supplemental Table S1:** Primer sequences, PCR conditions and sizes of PCR products corresponding to each CNE.

| **Segment** | **Primers** | Sequence of primer | **Annealing temperature** | **PCR Product (bp)** |
| --- | --- | --- | --- | --- |
| CNE-A | 1R  1F | GGTTCTGGGTGATGTTGAC GCCACAGTGCACTAAGACTATC | 59 | 562 |
| CNE-B | 1R  1F  2R  2F | TTTAATTTTGGTGCCTGCGTCAC  CTTCGCCTGCCTCCCTTCTAA  GGGCTGTGGGTCTAC  TCTCACAGCGTCGTTC | 62 | 465  228 |
| CNE-C | 1R  1F  2R  2F | ATGGTGCCAGATAGGTACTCAC  GCATTACAACAAATTCGGACA  GCTTTTTGCTTTGCATAGAAG  TATGGGTAAGGACCTTATGGAC | 61  65 | 562  335 |
| CNE-D | 1R  1F | CCCGGGATAACTAATGAAATGAGA GAGACTTCCTATGGCAACAGAAAAG | 63 | 585 |
| CNE-E | 1R  1F  2R  2F | CGGAAATTCAAAGGCTTGTTGTTG  TCACCCTGCCCACATAAAATACCA  CTTGGCATGCTGGTATTTTATGTG  GGAGGCATGGGGAGTGACTGATA | 57  57 | 396  464 |
| CNE-F | 1R  1F | CTTTCTGCTTTGGTCCTAATAA GGATCATTTGAGCCTAAGAGT | 64 | 837 |

Supplemental **Table S2**: Genotyping methods and samples genotyped for CNE polymorphisms.

| SNP | CNE | dbSNP rs# | Genotyping Method* | Sample(s) genotyped^+^ |
| --- | --- | --- | --- | --- |
| IL13_–1112C/T |  | 1800925 | DNAprint  LAS | HT  EA, AA |
| IL13_+1923C/T |  | 1295686 | LAS | HT, EA, AA |
| IL13_130A/G |  | 20541 | DNAprint | HT, EA, AA |
| IL4_–589C/T |  | 2243250 | LAS | HT, EA, AA |
| SNP2-C/T | CNE-C | 2070874 | AOD | HT, EA, AA |
| SNP4-C/T |  | 734244 | AOD | HT, EA, AA |
| IL4_+3017-G/T |  | 2227284 | AOD | HT, EA, AA |
| IL4_+8374A/G |  | 2243289 | DNAprint | HT, EA, AA |
| SNP7-G/A |  | 17539086 | DNAprint | HT, EA, AA |
| SNP8-C/G | CNE-F | 17539079 | SBE-FP^&^ | HT, EA, AA |

* Genotyping methods: DNAprint Genomics (Sarasota, FL) using a modified Orchard biosciences 25K/UHT hybrid system (<http://www.dnaprint.com/genotyping.html>); LAS, multiplex PCR and an immobilized linear array system [56, 57]; Assays-on-Demand (AOD) (Applied Biosystems, Foster City, CA; (<http://myscience.appliedbiosystems.com/genotype/search.jsp?assayType=genotyping>); SBE-FP, single-base extension with fluorescent polarization [58]

^+^ AA, African American; EA, European American; HT, Hutterites.

^&^ SBE-FP extension primers (annealing temperatures): SNP7: 5’-AAATCTTCCTACTTTCCTTTACCAT-3’ (59 °C); SNP8: 5’-AAGCGTGTTTGGGTGAGACAATCTGAT-3’ (55 °C)
